# Transcranial electrical stimulation (tES) mechanisms and its effects on cortical excitability and connectivity

**DOI:** 10.1007/s10545-018-0181-4

**Published:** 2018-07-13

**Authors:** Thomas Reed, Roi Cohen Kadosh

**Affiliations:** Department of Experimental Psychology, University of Oxford, New Richards Building, 71-73 Old Road, Oxford, OX3 7LA UK

## Abstract

In this review, we describe transcranial electrical stimulation (tES) techniques currently being used in neuroscientific research, including transcranial direct current (tDCS), alternating current (tACS) and random noise (tRNS) stimulation techniques. We explain how these techniques are used and summarise the proposed mechanisms of action for each technique. We continue by describing how each method has been used to alter endogenous neuronal oscillations and connectivity between brain regions, and we conclude by highlighting the varying effects of stimulation and discussing the future direction of these stimulation techniques in research.

## Introduction

**Fig. 1:**
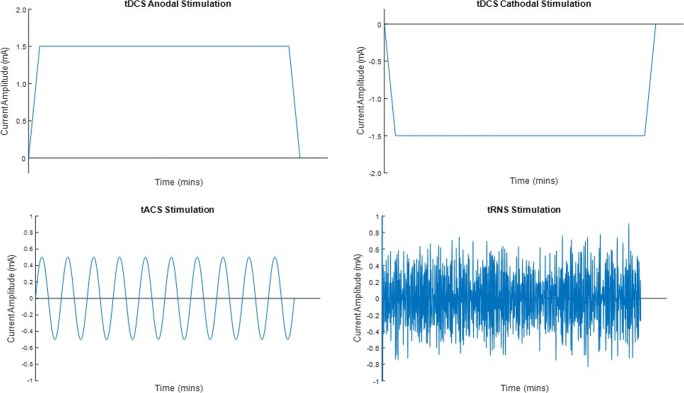
Examples of stimulation waveforms for transcranial direct current (tDCS) (anodal and cathodal), transcranial alternating current (tACS) and transcranial random noise (tRNS) stimulation

Transcranial electrical stimulation (tES) is a noninvasive brain stimulation technique that passes an electrical current through the cortex of the brain in to alter brain function. The electrical current is applied to an individual’s scalp usually via two or more electrodes, and whilst a large amount of the current is conducted between electrodes through soft tissue and skull (Vöröslakos et al. 2018), a portion of the current penetrates the scalp and is conducted through the brain, where it can alter neuronal excitability. By altering the activity of brain regions involved with a behaviour of interest, researchers can observe the resulting behavioural changes and so establish a causal link between the two. tES comprises a number of different techniques, including transcranial direct current stimulation (tDCS), alternating current stimulation (tACS) and random noise stimulation (tRNS) (Fig. 1). Whilst these techniques are similar in that they are applied through electrodes placed on the scalp, ES patterns, and therefore behavioural and neuronal outcomes, differ. Crucially, in contrast to another commonly used brain stimulation techniques called transcranial magnetic stimulation (TMS), the current delivered in tES techniques is not powerful enough to elicit an action potential and is maintained at subthreshold levels to effect cortical excitability only (Radman et al. 2009). In this article, we discuss each technique and demonstrate how they alter neuronal oscillations and connectivity between different brain regions.

## Applying tES

When employing tES, researchers commonly use two conductive rubber electrodes placed in saline-soaked sponges and attach at least one of them to the head with nonconductive elastic straps. The location of the active electrode (anode) depends on the cortical area to be modulated, whilst the return electrode (cathode) is usually placed in an area unrelated to the brain processes being examined, such as the forehead or vertex, but could also be placed on an extracephalic location. Although the most common electrode size used is 20–35 cm^2^ (Moreno-Duarte et al. 2014), this can also be modified to suit the needs of the researcher, with a more recent development being the high-definition stimulation, which uses arrays of smaller electrodes to give a more focalised stimulation area (Dmochowski et al. 2011; Edwards et al. 2013) or smaller electrodes (e.g. Phi electrodes). Precise electrode placement is normally derived from the International electroencephalography (EEG) 10–20 system [see Woods et al. (2016) for a comprehensive guide in administering tES].

## Transcranial direct-current stimulation

tDCS uses a direct current delivered at low intensities (e.g. 0.5–2 mA) through one or more active electrodes (anode). This current then propagates through the head and is returned via the reference electrode (cathode). This one-way flow of electricity reliably modulates cortical excitability (Nitsche and Paulus 2000), with excitability usually increasing at the anodal electrode (Boros et al. 2008) and decreasing at the cathodal electrode (Ardolino et al. 2005). This phenomenon has been clearly demonstrated using an anodal tDCS over the motor cortex to increase the amplitude of motor-evoked potentials (MEPs) and cathodal tDCS to decrease MEP amplitude (Paulus 2011). Importantly, the low-intensity electrical field generated by tDCS is subthreshold, meaning that it is capable of modifying neuronal transmembrane potentials and modulating excitability, thereby bringing the underlying neurons closer to their firing threshold without eliciting depolarisation (Bikson et al. 2004). Numerous studies have investigated the mechanisms of tDCS, Specifically, tDCS has been shown to act by decreasing γ-aminobutyric acid (GABA) concentrations (Stagg et al. 2011; Bachtiar et al. 2015) and increasing brain-derived neurotrophic factor (Fritsch et al. 2010) and glutamate and glutamine concentrations (Hunter et al. 2015). N-methyl-D-aspartate (NMDA) receptors also play a key role, with both short- and long-term effects of tDCS not being observed after blocking Na^+^ channels or after administration of an NMDA receptor antagonist (Liebetanz et al. 2002; Nitsche et al. 2003).

## tDCS: neural oscillations and connectivity

As changes in neural oscillations have been found in all major neurological diseases (Buzsáki and Watson 2012), it is of vital importance that researchers and clinicians have a method of modulating such oscillations to both research and potentially treat neurological conditions (Zich et al. 2017). Whilst tDCS is unable to target specific frequencies of oscillations, as previously mentioned, it is able to alter excitability and therefore neuronal activity in particular brain regions (Nitsche and Paulus, 2000). Interestingly, tDCS also decreases GABA in resting-state networks (Bachtiar et al. 2015), which has in turn been linked to changes in resting-state connectivity (Bachtiar et al. 2015; Stagg et al. 2014). Indeed, a number of studies demonstrate that tDCS is capable of altering connectivity. For example, Keeser et al. (2011) investigated whether tDCS can alter resting-state network connectivity by exposing participants to real or sham stimulation in two different sessions during which the anode was placed over the left dorsal lateral prefrontal cortex (DLPFC) and the cathode over the right supraorbital region. Participants received 20 min of 2 mA real or sham stimulation, and functional magnetic resonance imaging (fMRI) resting-state data were recorded before and after stimulation. When compared with sham, real tDCS participants showed significant changes in regional brain connectivity in the default mode network and frontal–parietal networks. These results clearly demonstrate the ability of tDCS to modulate resting-state connectivity. Further, Polanía et al. (2012) examined the differential effects of anodal and cathodal stimulation delivered over the motor cortex and demonstrated an increase within corticostriatal and thalamocortical circuits in response to anodal stimulation and a decrease in connectivity in response to cathodal stimulation, confirming the different effects of the two electrodes on connectivity. In another similar study, Polanía et al. (2011) demonstrated that tDCS can also modulate connectivity by not only increasing communication between areas related to the task, but also by reducing communication between other areas. In addition, they showed that changes in connectivity were higher during the motor task than at rest. Task-related connectivity changes have been demonstrated in a wide variety of other tasks, including risk taking (Weber et al. 2014), on a sensorimotor rhythm brain computer interface task (Baxter et al. 2017), a smoking cue reactivity task (Yang et al. 2017) and during speech (Holland et al. 2016). Examination of glutamatergic neurotransmission during anodal tDCS using proton magnetic resonance spectroscopy (MRS) found that glutamate and glutamine concentrations (Glx) were increased under the anodal electrode, and individual differences in Glx predicted network connectivity (Hunter et al. 2015) and remote effects on brain regions that were not directly beneath the electrodes (Hone-Blanchet et al. 2016).

## Transcranial alternating-current stimulation

tACS uses an electrical current that alternates between electrodes, usually in a sinusoidal wave. Unlike tDCS, tACS does not alter neuronal excitability but entrains the neuronal firing from the large number of underlying neurons to the exogenous frequency (Battleday et al. 2014). Neuronal entrainment is achieved by the applied current altering the transmembrane potential of neurons. Polarisation of neurons reflects the current applied to it, leading to a sinusoidal fluctuation of the membrane potential. As this fluctuation is both frequency dependent and linearly proportional to the applied current, lower-frequency stimulation induces larger polarisation than does higher frequencies (Reato et al. 2010) [see Tavakoli and Yun (2017) for a full description of tACS]. The ability to entrain neurons in a specific brain region to fire at a predetermined frequency enables researchers to identify the key frequencies involved in different behaviours and to draw causal links between them (Fig. 1).

## tACS: neural oscillations and connectivity

The ability of tACS to target and entrain specific frequencies within the brain is an invaluable tool for researchers and clinicians alike. Numerous studies have confirmed the ability of tACS to selectively entrain neural oscillations (Helfrich et al. 2014; Thut et al. 2011; Zaehle et al. 2010), even with a short duration of stimulation (Stonkus et al. 2016). Additionally, entrainment is at its most effective when tACS is applied at the same frequency as endogenous oscillations (Fröhlich and McCormick 2010; Reato et al. 2010). This observation suggests that tACS stimulation is brain-state dependent, which is supported by work by a number of studies (Alagapan et al. 2016; Neuling, Rach, and Herrmann, 2013), including Violante et al. (2017), who demonstrated this using a working memory task where they found that tACS modulated activity when cognitive demands were high. As with tDCS, tACS has been shown to alter local GABA levels (Nowak et al. 2017), specifically, GABA_A_, suggesting it could modulate connectivity (Stagg et al. 2014); however, few studies have reported a change in connectivity in response to tACS. Weinrich et al. (2017) delivered tACS to the primary motor cortex at 20 Hz, 5 Hz, or sham during three fMRI scans and found a significant change in the connectivity pattern of the primary motor cortex, without finding a change in the local activity or network connectivity. Additionally, in a similar experiment, Bächinger et al. (2017) stimulated participant’s left and right sensorimotor cortices at their endogenous alpha rhythm (8-12 Hz). The authors found an increase in connectivity between areas. and reported that this effect outlasted the stimulation period and tended to be more effective in individuals who exhibited a naturally weak interhemispheric coupling.

## Transcranial random noise stimulation

tRNS is a relatively recently developed tES method, being originally used in human participants in 2008 (Terney et al. 2008). tRNS is similar to tACS in that it uses an alternating current; however, instead of stimulating at a fixed frequency throughout the stimulation period, tRNS alternates at a random frequency and amplitude within a specific range. In a comparison of tDCS, tACS and tRNS, one study showed that tRNS is the most effective tES method for increasing cortical excitability of the motor cortex (Inukai et al. 2016). When using tRNS, stimulation frequency is normally distributed between 0.1 and 640 Hz (Terney et al. 2008), although it can be divided into either low- (0.1–100 Hz) or high- (101-640 Hz) frequency stimulation (Fertonani et al. 2011). It has clear neuronal and behavioural effects, with 10 min of stimulation increasing motor cortex excitability for ~1 h (Terney et al. 2008), although a study by Campana et al. (2016) suggests that low- and high-frequency tRNS can have opposing effects on cortical excitability. Indeed, Moliadze et al. (2012) reported findings showing that these excitability changes induced by tRNS are intensity dependent, with lower intensities (0.4 mA) eliciting inhibition and higher intensities (1 mA) eliciting excitation. Whilst the authors are uncertain as to the reason for this reversal in cortical excitability at lower intensities and indicated this finding needs to be replicated, they suggest that lower-intensity stimulation was able to either selectively facilitate intracortical inhibitory networks or inhibit intracortical facilitatory networks on corticospinal motor neurons. Shorter durations of tRNS have been used to alter cortical excitability (Chaieb et al. 2011); however, different durations of stimulation or combinations with tasks can result in different outcomes of stimulation (Chaieb et al. 2009), suggesting that shorter durations may not give as reliable results.

Whilst the mechanisms behind tRNS are not clearly understood in humans, in the rat, periods of repetitive high-frequency stimulation cause inward sodium currents within the neuron and weak depolarisation (Schoen and Fromherz 2008). Building on this work, Chaieb et al. (2015) showed that in humans, the excitability enhancing effects of tRNS are significantly decreased by blocking voltage-gated sodium channels. Using a combination of central nervous system (CNS) active drugs and single-pules TMS, the effects of tRNS are likely to be independent of NMDA receptors (Chaieb et al. 2015), indicating a different mechanism of action from tDCS (Liebetanz et al. 2002; Nitsche et al. 2003). Based on results from physiological and pharmacological studies, several theories explaining the mechanisms behind tRNS have been suggested. One proposed theory is stochastic resonance, whereby tRNS induces random activity within the target neurons (noise), which boosts sensitivity of the neurons to further external inputs (Miniussi et al. 2013; van der Groen and Wenderoth 2016). Alternatively, Fertonani et al. (2011) suggest that the mechanism of action of tRNS is based on repeated subthreshold stimulations, which may prevent homeostasis of the system and potentiate task-related neural activity.

## tRNS: neural oscillations and connectivity

To date, comparatively few studies have been published examining the effects of tRNS. The technique’s effects on cortical excitability, especially in the motor cortex, have been examined, although one study used near-infrared spectroscopy to document change in cortical excitability in the prefrontal cortex during arithmetic training (Snowball et al. 2013). However, only two papers have been published investigating changes in EEG measures. Van Doren et al. (2014) investigated tRNS-induced changes in resting-state activity and found a trending increase in theta power in frontal and parietal regions in response to 20 min of 2 mA offline stimulation delivered over the auditory cortex. In another similar study investigating tRNS of the auditory cortex, Rufener et al. (2017) found a limited effect on auditory event-related potentials. At the time of writing, no papers had been published investigating connectivity changes associated with tRNS.

## Variation in stimulation

Whilst the potential to modulate cognitive processes such as connectivity using tES techniques is clearly a reality, attention must be paid to factors affecting the results of stimulation. Specifically, when it comes to brain stimulation, variation between individuals is key, as not only can the optimal balance between cortical excitation and inhibition vary between individuals (Krause et al. 2013), but a large proportion of individuals fail to respond to stimulation altogether (López-alonso et al. 2014). Age (Leach et al. 2018), gender (Russell et al. 2017), tissue composition under the stimulating electrodes (Russell et al. 2013), and other factors (Krause and Cohen Kadosh 2014) have been suggested to alter the current density of stimulation or the elicited behavioural effects. In addition, stimulation results can be significantly altered by a large number of methodological decisions, including electrode placement (Moliadze et al. 2010), current intensity (Bastani and Jaberzadeh 2013) and, in the case of tACS and tRNS, current phase and frequency (Nakazono et al. 2016).

## Safety and tolerability of tES

A review of the adverse effects associated with tDCS in over 33,200 sessions and 1000 individuals reported that no serious adverse effects (severe or medically significant events) have been recorded whilst using tDCS (Bikson et al. 2016). Moderate adverse effects, such as skin burning due to poor electrode–skin contact, have been rarely reported, and mild adverse effects, such as skin irritation, headaches and fatigue, are frequently reported but seen in both active and sham stimulation (Bikson et al. 2016). Additionally, tACS and tRNS induce less sensation than tDCS (Fertonani et al. 2015). When using tES in a research or clinical setting, precautions are usually taken to prevent serious or moderate adverse effects from occurring; the duration (<60 min) and intensity (<4 mA) of stimulation as well as electrode size and placement is carefully selected to avoid increasing the temperature under the electrodes to prevent skin burns and limit any irritation (Antal et al. 2017). The skin is also prepared by cleaning with alcohol or a mildly abrasive scrub to remove any dirt or oils that may reduce conductivity and increase sensation. A comprehensive guide to the safety considerations surrounding tES use has been published by Antal et al. (2017) as the result of a 2-day conference on the safety of tES methods. The conference involved leading researchers, clinicians and manufacturers of stimulating devises, and the interested reader is referred to this publication.

## Ethical considerations

Whilst there is considerable evidence for the benefits of tES, particularly when combined with behavioural training paradigms (Krause and Cohen Kadosh 2013; Santarnecchi et al. 2015), there are still a number of ethical considerations that must be taken into account. A key area for scrutiny is the potential for unknown long-term changes in cortical function and behaviour. As the long-term effects of tES cannot always be guaranteed, the potential for inducing undesirable long-term effects in participants with or without fully informed consent is a real possibility. With the relatively minimal costs involved in acquiring a stimulating device (as little and £500), as well as the ease at which a device can be made using off-the-shelf components (Fitz and Reiner, 2014), tES can increasingly be applied by novice users or do-it-yourself (DIY) brain-stimulation enthusiasts. This raises the concern that it may be tried on vulnerable patient groups as a potential “improve-all” technique for cognitive enhancement without user knowledge of the ideal stimulation protocols or possible adverse side effects (Cohen Kadosh et al. 2012; Maslen et al. 2014). Additionally, stimulation parameters may not be kept within the safety guidelines, and stimulation sites may be misidentified, causing stimulation to affect different cognitive processes than those intended, leading to a decline in already worsened cognitive abilities (for a full reviews of DIY-tES, see Fitz and Reiner 2014; Hamilton et al. 2011).

## Future directions of tES in research

Whilst the area of tES research has advanced a great deal within the past decade, further advancements must be made if the techniques are to be used to their full potential. As discussed previously, the mechanisms underlying stimulation are not fully understood despite multiple studies attempting to characterise it. As this paper highlights, very little is known about tRNS in particular, with several mechanistic theories proposed, and very few studies have investigated the cortical responses to tRNS. Further work to characterise the effects of all stimulation types at the behavioural and neuronal levels are needed to fully elucidate their mechanisms of action. 

Also discussed in this article is the issue of individual variability in response to stimulation, an important subject in any methodological technique but one shown to be crucial in brain stimulation. Several research groups are undertaking work targeting this very issue, with two main approaches being taken: either by selecting an individual basis for stimulation type (tDCS/tACS/tRNS) that shows the most potential for behavioural improvements or neuromodulation, or by identifying the optimal stimulation parameters, such as electrode location or current for a specific type of stimulation, that are most likely to have the desired effect in each individual. In addition, recent studies (Alagapan et al. 2016) have tried to reduce intra-individual variability by accounting for the brain state of the individual, which is a clear factor in outcomes from stimulation (Neuling et al. 2013). Such work would allow not only better mechanistic understanding but also progression towards the design of closed-loop systems to stimulate individuals at specific brain states without input from the experimenter, as has been done using invasive stimulation techniques (Ezzyat et al. 2018).

As is clear, future work must be aimed at understanding the mechanisms of stimulation and improving stimulation outcomes. Currently, tES experiments examine its future use in different neurological conditions, including stroke (O’Shea et al. 2014), Alzheimer’s disease (Boggio et al. 2011) and Parkinson’s disease (Rektorová and Anderková 2017). With greater understanding of the underlying mechanisms, application of tES to disease is likely to become ever more prominent in the future and could unlock key therapies for individuals suffering from neurological conditions.
